# Defining and analysing symptom palliation in cancer clinical trials: a deceptively difficult exercise

**DOI:** 10.1038/sj.bjc.6690085

**Published:** 1999-02

**Authors:** R J Stephens, P Hopwood, D J Girling

**Affiliations:** MRC Cancer Trials Office, 5 Shaftesbury Road, Cambridge, CB2 2BW, UK; CRC Psychological Medicine Group, Stanley House, Christie Hospital NHS Trust, Withington, Manchester, M20 4BX, UK

**Keywords:** symptom palliation, clinical trials

## Abstract

The assessment of symptom palliation is an essential component of many treatment comparisons in clinical trials, yet an extensive literature search revealed no consensus as to its precise definition, which could embrace relief of symptoms, time to their onset, duration, degree, as well as symptom control and prevention. In an attempt to assess the importance of these aspects and to compare different methods of analysis, we used one symptom (cough) from a patient self-assessment questionnaire (the Rotterdam Symptom Checklist) in a large (>300 patient) multicentre randomized clinical trial (conducted by the Medical Research Council Lung Cancer Working Party) of palliative chemotherapy in small-cell lung cancer. The regimens compared were a two-drug regimen (2D) and a four-drug regimen (4D). No differences were seen between the regimens in time of onset of palliation or its duration. The degree of palliation was strongly related to the initial severity: 90% of the patients with moderate or severe cough at baseline reported improvement, compared with only 53% of those with mild cough. Analyses using different landmark time points gave conflicting results: the 4D regimen was superior at 1 month and at 3 months, whereas at 2 months the 2D regimen appeared superior. When improvement at any time up to 3 months was considered, the 4D regimen showed a significant benefit (4D 79%, 2D 60%, *P* = 0.02). These findings emphasize the need for caution in interpreting results, and the importance of working towards a standard definition of symptom palliation. The current lack of specified criteria makes analysis and interpretation of trial results difficult, and comparison across trials impossible. A standard definition of palliation for use in the analysis of clinical trials data is proposed, which takes into account aspects of onset, duration and degree of palliation, and symptom improvement, control and prevention. *© 1999 Cancer Research Campaign*


*British Journal of Cancer* (1999) **79**(3/4), 538–544
© 1999 Cancer Research Campaign
Article no. bjoc.1998.0085

### Defining and analysing symptom palliation in cancer clinical trials: a deceptively difficult exercise

**RJ Stephens[1], P Hopwood[2] and DJ Girling[1]**

[1]MRC Cancer Trials Office, 5 Shaftesbury Road, Cambridge CB2 2BW, UK; [2]CRC Psychological Medicine Group, Stanley House, Christie Hospital NHS Trust, Withington, Manchester M20 4BX, UK

**Summary** The assessment of symptom palliation is an essential component of many treatment comparisons in clinical trials, yet an extensive literature search revealed no consensus as to its precise definition, which could embrace relief of symptoms, time to their onset, duration, degree, as well as symptom control and prevention. In an attempt to assess the importance of these aspects and to compare different methods of analysis, we used one symptom (cough) from a patient self-assessment questionnaire (the Rotterdam Symptom Checklist) in a large (>300 patient) multicentre randomized clinical trial (conducted by the Medical Research Council Lung Cancer Working Party) of palliative chemotherapy in small-cell lung cancer. The regimens compared were a two-drug regimen (2D) and a four-drug regimen (4D). No differences were seen between the regimens in time of onset of palliation or its duration. The degree of palliation was strongly related to the initial severity: 90% of the patients with moderate or severe cough at baseline reported improvement, compared with only 53% of those with mild cough. Analyses using different landmark time points gave conflicting results: the 4D regimen was superior at 1 month and at 3 months, whereas at 2 months the 2D regimen appeared superior. When improvement at any time up to 3 months was considered, the 4D regimen showed a significant benefit (4D 79%, 2D 60%, $P = 0.02$). These findings emphasize the need for caution in interpreting results, and the importance of working towards a standard definition of symptom palliation. The current lack of specified criteria makes analysis and interpretation of trial results difficult, and comparison across trials impossible. A standard definition of palliation for use in the analysis of clinical trials data is proposed, which takes into account aspects of onset, duration and degree of palliation, and symptom improvement, control and prevention.

**Keywords:** symptom palliation; clinical trials

It is surprising that although many cancer treatment trials are palliative in intent, there is no agreed definition of ‘palliation’ or a standard approach to its measurement. Yet, if palliative treatments, whether they be for symptoms of the disease or side-effects of the cancer treatment, are to be compared, some precision in the assessment of treatment policies is essential. It is our concern that the reporting of symptom palliation is often oversimplified, and that in fact it is deceptively difficult to uncover the real effect of treatment on patient groups.

With the introduction of health-related quality of life (QL) questionnaires to measure longitudinal changes in a broad range of symptoms, it was expected that a more detailed evaluation of palliation would be feasible. The Medical Research Council (MRC) Lung Cancer Working Party (LCWP) has used such an instrument, the Rotterdam Symptom Checklist (RSCL) (De Haes et al, 1990), in successive trials. It has become apparent that the analyses of these data pose some formidable problems, partly because of high attrition rates and missing data (Hopwood et al, 1994) and partly because of the lack of an agreed definition of palliation. Indeed, the definition of the word ‘palliation’ and the level of complexity with which it is reported vary enormously.

*Received 11 March 1998*
*Revised 24 June 1998*
*Accepted 27 June 1998*

*Correspondence to:* RJ Stephens, MRC Cancer Trials Office, 5 Shaftesbury Road, Cambridge CB2 2BW, UK

### THE STATE OF THE ART: A REVIEW OF THE LITERATURE

In published reports, ‘palliative treatment’ may simply reflect non-curative treatment, with no information provided on relief of presenting symptoms [e.g. trials of palliative chemotherapy in small-cell lung cancer (James et al, 1996) and hormonal palliation of ovarian cancer (Ahlgren et al, 1993)]. In other reported trials, palliation is defined as the treatment given to prevent medical conditions arising [e.g. pamidronate to prevent hypercalcaemia and bone pain in patients with bone metastases (van Holten-Verzantvoort et al, 1993)].

When relief of symptoms is considered, the most straightforward analyses are those in which the primary end point is the resolution of a specific symptom [e.g. sucralfate to palliate acute radiation oesophagitis (Sur et al, 1994) or granisetron as an antiemetic in cytostatic-induced emesis (Marty et al, 1992)]. Occasionally, when treatment affects a number of symptoms, the individual symptoms are listed and their palliation reported [e.g. neurological symptoms in patients with brain metastases (Kurtz et al, 1981) or general symptoms resulting from hepatic metastases (Mohiuddin et al, 1996)].

When assessments have been made over several symptoms or time points, authors have used a variety of methods of simplifying analysis and presentation by summarizing the symptoms or the time points. For example, Lewington et al (1991) combined change in general condition, mobility, analgesic use and pain into a symptom response. In contrast, Barr et al (1990) compared the

**Table 1** Patient sample for analysis

| | 4D regimen (*n*) | 2D regimen (*n*) | Total (*n*) |
|---|---|---|---|
| Total patients | 154 | 156 | 310 |
| No baseline data | 16 | 29 | |
| Baseline completed too late (> 7 days) | 1 | 1 | |
| No follow-up RSCLs completed | 49 | 23 | |
| No cough at baseline | 17 | 17 | |
| Remaining for analysis | 71 | 86 | 157 (51%) |

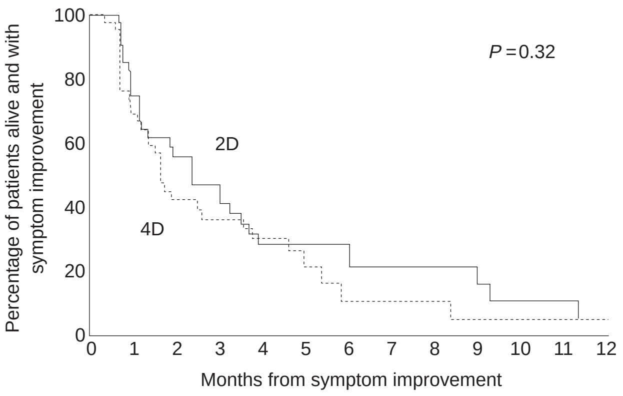

**Figure 1** Kaplan–Meier plot of duration of symptom improvement

'mean' and 'best' dysphagia score against baseline, and Ball et al (1997) calculated an average grade for each symptom.

All too often, results presented give scant information on how symptoms were measured, by whom, when, for how long, and what the severity was at baseline and thereafter (e.g. Haie-Meder et al, 1993; Buroker et al, 1994). Nevertheless, a number of groups have investigated more innovative ways of analysing palliation in the context of their trials.

Burris and Storniolo (1997) attempted to demonstrate 'clinical benefit', and categorized their patients as responders based on changes in pain (analgesia use or intensity), Karnofsky performance status and weight. Such an approach is potentially useful, but requires considerable preparatory work.

Tong et al (1982), investigating radiotherapy schedules to palliate symptomatic bone metastases, considered the promptness of pain relief. However, as the authors point out, the comparison was based not on when relief occurred, but when it was reported, which introduces the possibility of bias.

Tannock et al (1996), in a trial of chemotherapy for prostate cancer, devised a palliative response based on pain intensity and analgesic intake. They then used Kaplan–Meier plots to show the duration of palliation.

Leibel et al (1987) considered the palliation achieved within groups of patients who had different levels of initial severity. This indicated that a greater proportion of patients with moderate or severe abdominal pain reported relief after treatment than those with mild pain, but that complete response was less often achieved.

A palliation index in patients with lung cancer (Muers and Round, 1993) was based on the duration of response divided by the duration of survival. Importantly, this definition included the concept of symptom control, so that palliation encompassed patients with initially no symptoms or mild symptoms which did not get worse.

There is little evidence, however, that any of these potentially useful methods have been repeated by the authors or applied more widely.

The MRC LCWP have used a variety of methods of examining clinician-rated symptom relief in a series of trials of palliative radiotherapy in non-small-cell lung cancer (NSCLC) and palliative chemotherapy in small-cell lung cancer (SCLC). In three trials (MRC LCWP, 1991, 1992, 1993), the duration of improvement of individual presenting symptoms was calculated and then (as with Muers and Round, 1993) presented as a proportion of survival time. In addition, the proportion of patients achieving complete resolution of presenting symptoms and duration of palliation was reported, and better palliation in patients with initially moderate or severe symptoms was noted. In two further trials (MRC LCWP, 1996*a, b*), Kaplan–Meier plots were used to estimate the frequency of palliation of individual patient-reported symptoms by specified time points. Finally, in the most recently published trial (MRC LCWP, 1996*c*), a palliation score was devised to incorporate four key symptoms, and changes in this score from baseline to 3 months, importantly including patients who died before 3 months as non-palliated, were compared.

### AIM

The aim of this paper is to report exploratory analyses that highlight some of the advantages and disadvantages of published methods, and to try to develop a generally applicable definition of symptom palliation in clinical trials. To do this, we have used data from patient-completed RSCL questionnaires in an MRC LCWP trial because this is typical of the data available in many trials.

However, the concepts are relevant to any assessments of symptoms, whether they be by the patient or the clinician or on any standard QL questionnaire.

### DATA USED

Data from the MRC LCWP LU12 trial (MRC LCWP, 1996*b*) was used in this analysis. In the LU12 trial, patients with extensive disease SCLC, or limited disease but poor performance status [WHO grade 3 or 4 (WHO, 1979)], were allocated at random to receive three 3-weekly cycles of either a four-drug regimen – etoposide, cyclophosphamide, methotrexate and vincristine (4D) or a two-drug regimen – etoposide and vincristine (2D) as palliative treatment. The 4D regimen had been shown in previous MRC trials (MRC LCWP, 1989*a, b*) to be an effective treatment for SCLC, and the aim of this trial was to see if, in poor prognosis patients, the 2D regimen would prove equally effective but less toxic.

A total of 310 patients (154, 4D; 156, 2D) were randomized from 23 centres in the UK. There was no difference in survival between the two treatment groups (medians: 4D, 141 days; 2D, 137 days), and levels of palliation were reported to be similar, but patients on the 4D regimen had more haematological toxicity and more early, possibly treatment-related, deaths (Stephens et al, 1994).

### Assessment of Quality of Life

QL was assessed by patients using the RSCL, a patient-completed categorical scale consisting of 38 core items to which four lung cancer-specific symptoms (chest pain, cough, hoarseness and

*© Cancer Research Campaign 1999*

**Table 2** Improvement according to initial severity of cough

| Initial severity of cough | Regimen | Total patients | Not improved no. (%) | Improved to | | | Total (%) |
|---|---|---|---|---|---|---|---|
| | | | | Nil | Mild | Moderate | |
| Severe | 4D | 16 | 0 (0) | 8 | 4 | 4 | 16 (100) |
| | 2D | 18 | 3 (17) | 8 | 7 | 0 | 15 (83) |
| Moderate | 4D | 19 | 1 (5) | 8 | 10 | – | 18 (95) |
| | 2D | 15 | 3 (20) | 9 | 3 | – | 12 (80) |
| Mild | 4D | 36 | 14 (39) | 22 | – | – | 22 (61) |
| | 2D | 53 | 28 (53) | 25 | – | – | 25 (47) |

**Table 3** Improvement in severity of cough at different time points

| Time from randomization | Regimen | Patients assessed | Improvement no. | (%) | *P*-value |
|---|---|---|---|---|---|
| 1 month | 4D | 64 | 40 | (62) | |
| | 2D | 76 | 31 | (41) | 0.02 |
| 2 months | 4D | 36 | 18 | (50) | |
| | 2D | 50 | 30 | (60) | 0.50 |
| 3 months | 4D | 25 | 16 | (64) | |
| | 2D | 32 | 12 | (38) | 0.09 |

coughing up blood) were added. Patients were asked to tick boxes to indicate how they had been feeling during the past week. In this paper, these categories are referred to as nil, mild, moderate and severe. Questionnaires were completed pretreatment, at each attendance for protocol chemotherapy, then monthly to 6 months from randomization, 2 monthly to 1 year, and 3 monthly thereafter.

### Analyses

For the sake of simplicity, we have focused on one major disease-related symptom (cough).

Compliance was assessed according to the method proposed by Hopwood et al (1994), which gave an overall compliance of 56% in the first 6 months falling from 85% at baseline to 31% at 6 months. Such low levels of compliance, which are not unusual in palliative trials, affect the analysis of palliation. For example, to compare the treatments in terms of improving the severity of cough by one grade or more at any follow-up from baseline, Table 1 shows that, of the 154 4D and 156 2D patients randomized, 16 and 29, respectively, did not complete a baseline RSCL; a further one and one completed the form outside the specified time window; 49 and 23 completed no follow-up questionnaires; and 17 and 17 patients did not report having a cough at baseline. There thus remain 71 4D and 86 2D patients for any comparison of improvement of cough.

### Aspects of palliation

By synthesizing some of the ideas in the published papers, we propose that palliation can be viewed as a three-dimensional concept, namely, time of onset, duration and degree.

### Onset of symptom improvement

Considering the 157 patients with data available for analysis (Table 1), the majority of patients reported an almost immediate improvement, but 12 (11% of the patients who reported improvement) reported this later than 2 months from starting treatment. In one of these 12 patients, this was simply due to the absence of any earlier assessments, but the remaining 11 patients had intermediate assessments at which they reported no change or indeed a worsening cough.

When assessing time to onset of palliation, the timings and frequency of questionnaire administration and patient compliance are vitally important, and the time period over which palliation is to be assessed should be limited to that period when trial regimens, rather than additional interventions, will be influencing events.

One possible method of treatment comparison of the time of onset of symptom relief uses Kaplan–Meier plots: the time that improvement was first reported is taken as the event, with patients not reporting improvement being censored at the date of their last QL questionnaire completion (MRC LCWP, 1996*a*, *b*). The main advantage of this method is that all patients reporting cough at baseline can be included. The major disadvantage is that it assumes that censored patients [those not achieving the event (palliation) whether they be alive or dead] are assumed to follow the same pattern as those who do. Thus, a treatment with poor survival but good palliation among the survivors can appear better than a treatment with good survival and variable palliation, even if the actual number of patients alive and with palliation is exactly the same in both treatments. Similarly, any unequal reduction in group sample sizes must be considered worrying, e.g. Buroker et al (1994), from initial group samples of 183 and 179, compared 122 with 90 in terms of palliation.

If time to onset is too difficult a concept to include in an overall definition of palliation, at least limiting the period over which palliation is assessed ensures that it is related to the treatment being studied.

### Duration of improvement

Kaplan–Meier plots can be used to estimate and compare the proportion of patients who remain alive and with symptom improvement because it is reasonable to assume that patients censored will die or relapse at the same rate as their contemporaries. Figure 1 is based on 56 4D and 52 2D patients who reported improvement in cough. There was no statistically significant difference between the regimens ($P = 0.32$) using this method of comparison.

Of course, the accuracy of these figures relates to the frequency of assessment. The dates of symptom improvement and deterioration have to be taken as the date of the assessment at which the event was first reported, which could be weeks or even months after the previous assessment and after the actual event.

 © Cancer Research Campaign 1999

**Table 4** Analysis of palliation of cough, based on different definitions of palliation

| | | | | Early deaths included in the denominator | |
|---|---|---|---|---|---|
| **Definition of palliation** | **Regimen** | **Patients palliated/ assessed (%)** | ***P*-value** | **Patients palliated/ assessed (%)** | ***P*-value** |
| (i) Improvement at one or | 4D | 56/71 (79) | | 56/92 (61) | |
| more assessments | 2D | 52/86 (60) | 0.02 | 52/97 (54) | 0.39 |
| (ii) Disappearance at one or | 4D | 38/71 (54) | | 38/92 (41) | |
| more assessments | 2D | 42/86 (49) | 0.67 | 42/97 (43) | 0.90 |
| (iii) Improvement at two | 4D | 33/58 (57) | | 33/82 (40) | |
| successive assessments | 2D | 27/65 (42) | 0.13 | 27/78 (35) | 0.58 |
| (iv) Improvement of moderate | 4D | 30/35 (86) | | 30/47 (64) | |
| or severe to mild or nil at | 2D | 27/33 (82) | 0.92 | 27/37 (73) | 0.51 |
| one or more assessments | | | | | |
| (v) Improvement of moderate or severe to mild or nil at one | | | | | |
| or more assessments, | 4D | 62/88 (70) | | 62/121 (51) | |
| and mild or nil remaining | 2D | 66/103 (64) | 0.44 | 66/115 (57) | 0.41 |
| at mild or nil at all assessments | | | | | |

One simple way of incorporating duration into any definition of palliation is to ensure that the palliation reported is sustained, not transient or due to day-to-day changes. Thus, as with the WHO definition of tumour response (WHO, 1979) and as used by Tannock et al (1996), improvement should only be accepted if it is reported on two successive assessments at least 3 weeks apart.

### Degree

The degree of symptom relief was analysed according to the severity at presentation. Improvement in severity of cough was related to the reported baseline severity (as shown in Table 2). Thus, in the 4D group, of the patients who reported severe cough at baseline, all 16 (100%) reported improvement, compared with 18 (95%) of those who reported moderate cough at baseline and only 22 (61%) who reported a mild cough. The corresponding figures for the 2D group were 83%, 80% and 47%.

Therefore, different criteria for ‘palliation’ are required for patients presenting with different symptom severity. These can be referred to as improvement, control and prevention.

### *Improvement*

The intuitive definition of palliation is that the symptom is relieved. Given the wide use of a four-point categorical severity scale, this is inevitably defined as an improvement in symptom rating by one or more categories (Sur et al, 1994; MRC LCWP, 1991, 1992, 1993, 1996*a–c*). Inevitably, patients without the symptom at presentation are excluded.

### *Control*

As suggested by Muers and Round (1993), for those patients with absent or mild symptoms at presentation it makes clinical sense to consider control, i.e. the symptom does not develop or, if mild, get worse.

### *Prevention*

Another way of including the patients reporting no initial symptoms is to include prevention as part of palliation (e.g. van Holten-Verzantvoort et al, 1993).

Thus, all patients can be encompassed in a definition which requires improvement in those with initially moderate or severe symptom, improvement or control for those with mild symptom, and prevention in those with no symptom. This accords with the definition of palliation outcome measures described by Maher et al (1994).

### SELECTING THE TIME POINT FOR ANALYSIS

The landmark analysis, calculating the change from baseline to a specific time point, is most often used in the analysis of palliation. However, there are two distinct methods, both with inherent problems. Using all the available data results in a diminishing sample [e.g. Twelves et al (1994) compared 14 patients at baseline with 12 at 6 weeks], which is not strictly comparable and does not account for the fact that any improvement seen may simply be due to patients who are more ill dropping out. Alternatively, using only those patients with data at both time points does not allow for the fact that such patients, who must be survivors and compliers, may not be representative of the whole sample, e.g. Brewster et al (1995) could use only 161 patients with data at baseline and at 6 weeks out of their sample of 197, and Richards et al (1992) could use only 42 out of 53 patients at pre- and post-treatment.

Our paper discussing this problem (Hopwood et al, 1994) suggests that, in order to utilize all the data, it is first necessary to show that the scores from patients completing different numbers of questionnaires (for whatever reason) are similar. Glimelius et al (1996) produced charts to show similar patterns of symptom severity in subgroups of patients within each regimen, and more importantly differences between regimens. However, as there are no simple statistical methods to compare plots composed of diminishing populations over time, such comparisons can only be descriptive. Thus, using only those patients with data at both baseline and the landmark time point is the preferred option.

In Table 3, three time points were considered: 1, 2 and 3 months from randomization. For each, the denominator was the number of patients with a reported cough at baseline and follow-up data at that specific time point, and the proportion of patients is reported whose grade of cough improved compared with baseline.

© Cancer Research Campaign 1999 

**Table 5** Proposed definition of palliation (based on two successive assessments in the first 3 months)

| | 4D regimen (*n*) | 2D regimen (*n*) | |
|---|---|---|---|
| Total patients | 154 | 156 | |
| Excluded from the analysis | | | |
| No baseline data | 16 | 29 | |
| Baseline completed >7 days after randomization | 1 | 1 | |
| Patients with inadequate follow-up data | 33 | 37 | |
| Remaining for the analysis | 104 | 89 | |
| | **Palliated/assessed (%)** | **Palliated/assessed (%)** | |
| Improvement (from moderate or severe to mild or nil) | 16/26 (62) | 12/22 (55) | |
| Control (mild, not getting worse) | 24/29 (83) | 29/40 (72) | |
| Prevention (nil, not getting worse) | 5/12 (42) | 8/13 (62) | |
| Subtotal | 45/67 (67) | 49/75 (65) | $P = 0.96$ |
| Died without palliation | 37 | 14 | |
| Total | 45/104 (43) | 49/89 (55) | $P = 0.14$ |

At 1 month and at 3 months, the 4D regimen emerged superior (and at 1 month significantly so, $P = 0.02$), whereas at 2 months the 2D regimen appeared favourable. The selection of time point appears critical, and it is very concerning to find that conflicting results can be obtained.

Considering several time points results in multiple comparisons and the need to adjust the level of statistical significance, therefore, reporting improvement at any one assessment over a specified period of time is probably more reliable than choosing one landmark time point.

### ANALYSIS OF PALLIATION: EXAMPLES OF METHODS IN USE

Five different definitions of palliation, each of which is commonly reported or presented, were applied to the MRC data set, and the proportions of patients reporting palliation of cough compared. The results are summarized in Table 4. In addition, to avoid the possibility that differences in survival biased results, for each analysis we added a subsidiary analysis in which the patients excluded because of early death were included as non-palliated (MRC LCWP, 1996*c*).

(i) Improvement from baseline by one or more categories at any subsequent assessment.

This is the most commonly used definition (MRC LCWP, 1991, 1992, 1993).

The population for this analysis are the 157 patients who reported having a cough at baseline and had at least 1 follow-up assessment (see Table 1). If the score for cough on any of the follow-up assessments is lower than that reported at baseline, that patient is considered to have been palliated. The analysis suggests a benefit for the 4D group (4D, 79%; 2D, 60%; $P = 0.02$).

However, if the patients who reported having a cough at baseline, but who died before completing the first follow-up QL questionnaire are added to the denominator, then this difference disappears: 4D, 61%; 2D, 54%; $P = 0.39$.

(ii) Disappearance of symptom at any subsequent assessment

In some circumstances, the aim of treatment is to eradicate symptoms completely. Using the same 157 patients as in definition (i), in this case only those who reported ‘nil’ at any subsequent assessment were considered palliated. The 4D regimen again emerged as superior to 2D (4D, 54%; 2D, 49%), but not statistically significantly so.

Adding in the patients who reported cough at baseline, but who died, to the denominator changed the apparent outcome, with the 2D regimen now achieving 43% palliation and the 4D achieving 41%.

(iii) Improvement from baseline by one or more categories at two successive assessments.

In an attempt to ensure that palliation obtained is clinically beneficial and not merely transient, palliation may be defined as symptom improvement sustained over two successive follow-up assessments (Tannock et al, 1996). In our data, the study population was reduced from the 157 used in definitions (i) and (ii) to 123 (58, 4D; 65, 2D), as 34 patients (13 and 21 respectively) only had one follow-up QL questionnaire assessment and were excluded. The benefit appeared to be associated with the 4D regimen: 4D, 57% palliated; 2D, 42%; $P = 0.13$.

In addition to the 21 and 11 patients, respectively, who reported a cough at baseline but who died before the first follow-up assessment and, therefore, can be classed as ‘not palliated’, we can also include three and two patients, respectively, who reported a cough at baseline, completed a QL questionnaire at first follow-up, but died before the

 © Cancer Research Campaign 1999

second follow-up. Adding these patients into the denominator gives palliation rates of 40% and 35% for the 4D and 2D patients respectively, $P = 0.58$.

(iv) Improvement from baseline of moderate or severe to mild or nil at any assessment.

There may be situations in which the aim of palliative treatment is to concentrate on patients with more severe symptoms. In this definition, only those with moderate or severe cough at presentation are considered, and only those who report this reduced to mild or nil are 'palliated'. In our dataset, the initial sample size was thus reduced from 157 patients to 68. The level of palliation achieved with this definition appeared higher than with previous definitions (4D, 86%; 2D, 82%) because, as already shown, of improvement being easier to demonstrate in patients with moderate or severe symptoms. This analysis suggests that there is no significant difference between the regimens.

Using this definition, we can add in the 16 patients (12, 4D; 4, 2D) who reported a moderate or severe cough at baseline and who died before the first follow-up assessment. This, as with definition (ii), suggests a non-significant benefit from the 2D regimen (4D, 64%; 2D, 73%).

(v) Improvement from baseline of moderate or severe to mild or nil, or no deterioration in mild or nil.

Other groups have suggested that the aim of palliation is not merely to relieve, but to control and to prevent symptoms. One major benefit of this definition is that it allows the inclusion of 34 patients (see Table 1), who reported no cough at baseline assessment but completed at least one follow-up questionnaire. Thus, those patients whose cough was moderate or severe at baseline must improve to nil or mild at some point (definition iv), and those with nil or mild at baseline must not get worse at any subsequent assessment. The analysis shows a non-significant benefit from the 4D regimen (4D, 70%; 2D, 64%).

In addition to the 32 patients (21, 4D; 11, 2D) who reported a cough at baseline but died before their first follow-up, we can also include the 13 (12, 4D; 1, 2D) who reported no cough at baseline, but who died. This suggests a slight benefit from the 2D regimen (4D, 51%; 2D, 57%).

### Summary

It is reassuring that, if the early deaths are not included as non-palliated, for all five definitions the same regimen emerged as the more effective of the two. However, the level of palliation, the size of the difference and the associated *P*-values varied widely. Clinicians' opinions of the palliative capacity of regimens, as well as the importance that they are likely to attach to differences between regimens, could therefore depend to a considerable extent on the definition used.

However, when account was taken of early deaths, by scoring these patients as 'not palliated' the results were inconsistent, and, although never reaching statistical significance, the benefit tended to favour the 2D regimen.

### APPLICATION OF THE PROPOSED DEFINITION OF PALLIATION

Because of the observed inconsistencies, we believe it is necessary to encompass improvement, control, prevention, degree, duration and onset. The results of this analysis are shown in detail in Table 5. The period of assessment was limited to the first 3 months from randomization, patients with moderate or severe cough at baseline were required to show improvement (a reduction to nil or mild on two successive assessments), those with mild cough at baseline to show control (not getting worse), and those with no symptoms to show no deterioration (prevention). To be included in this analysis, therefore, patients had to have completed questionnaires at baseline and at two successive follow-up assessments in the first 3 months, and those who died before the second follow-up but with complete data were included as 'not palliated'.

Using these criteria, a total of 117 patients (38%) were excluded, mainly because of lack of data, leaving 104 4D and 89 2D patients. Of the patients who started with moderate or severe cough, this improved to no or mild cough in 62% of the 4D patients and 55% of the 2D patients. Of the patients who started with a mild cough, this either remained mild or improved to nil in 83% 4D and 72% 2D. Finally, of the patients who started treatment without a cough, this did not develop in 42% 4D and 62% 2D. In total, therefore, 67% 4D and 65% 2D were considered to be palliated using this definition.

Adding in as 'not palliated' those patients who died, the balance shifts towards the 2D regimen, with 43% 4D and 55% 2D achieving palliation.

### CONCLUSION

Symptom palliation is likely to be a major end point in cancer therapy trials when it is unrealistic to aim for cure, as well as in other comparisons of treatments aimed at symptom relief or control (e.g. analgesics, antiemetics). Clinicians would benefit greatly from being able to compare published trial results and extract clinically relevant outcomes to discuss treatment decisions with their patients. Agreeing a definition of palliation in this context is an essential next step, and one that warrants urgent consideration given the amount of QL data currently being generated in palliative treatment trials.

For simplicity in our analyses, we used a single symptom, cough. In analysing the QL aspects of a clinical trial, it would be unwise, because of the dangers of multiple comparisons, as well as unwieldy, to present the results of every individual item. Nevertheless, we would also argue that combining items into subscales or domains can sometimes mask important differences in individual symptoms. The recommended approach is to set out predefined hypotheses, which also will have the advantage of helping to choose the correct questionnaire, deciding on the timing of administration, and of calculating a suitable sample size.

We recognize that our methods are not exhaustive or necessarily the best, but our aim has been to draw attention to the problems inherent in assessing palliation, to tease out the advantages and disadvantages and make some preliminary recommendations.

### ACKNOWLEDGEMENTS

We would like to thank the MRC LCWP (members: NM Bleehen, JJ Bolger, PI Clark, CK Connolly, DJ Girling, PS Hasleton, P Hopwood, FR Macbeth, D Machin, K Moghissi, MI Saunders, RJ Stephens, N Thatcher, RJ White) for allowing us the use of these data, and all the clinicians who entered patients into the LU12 trial as well as staff in the centres responsible for the collection of QL data.

© Cancer Research Campaign 1999

### REFERENCES

Ahlgren JD, Ellison NM, Gottlieb RJ, Laluna F, Lokich JJ, Sinclair PR, Veno W, Wampler GL, Yeung K-Y, Alt D and Fryer JG (1993) Hormonal palliation of chemoresistant ovarian cancer: three consecutive phase II trials of the Mid-Atlantic Oncology Program. *J Clin Oncol* **11**: 1957–1968

Ball D, Smith J, Bishop J, Olver I, Davis S, O'Brien P, Bernshaw D, Ryan G and Millward M (1997) A phase III study of radiotherapy with and without continuous-infusion fluorouracil as palliation for non-small cell lung cancer. *Br J Cancer* **75**: 690–697

Barr H, Krasner N, Raouf A and Walker RJ (1990) Prospective randomised trial of laser therapy only and laser therapy followed by endoscopic intubation for the palliation of malignant dysphagia. *Gut* **31**: 252–258

Brewster AE, Davidson SE, Makin WP, Stout R and Burt PA (1995) Intraluminal brachytherapy using the high dose rate microselectron in the palliation of carcinoma of the oesophagus. *Clin Oncol* **7**: 102–105

Buroker TR, O'Connell MJ, Weiand HS, Krook JE, Gerstner JB, Mailliard JA, Schaefer PL, Levitt R, Kardinal CG and Gesme Jr DH (1994) Randomized comparison of two schedules of fluorouracil and leucovorin in the treatment of advanced colorectal cancer. *J Clin Oncol* **12**: 14–20

Burris H and Storniolo AM (1997) Assessing clinical benefit in the treatment of pancreas cancer: gemcitabine compared to 5-fluorouracil. *Eur J Cancer* **33** (suppl. 1): S18–S22

De Haes JCJM, van Knippenberg FCE and Neijt JP (1990) Measuring psychological and physical distress in cancer patients: structure and application of the Rotterdam Symptom Checklist. *Br J Cancer* **62**: 1034–1038

Glimelius B, Hoffman K, Sjoden PO, Jacobsson G, Sellström H, Enander L-K, Linné T and Svensson C (1996) Chemotherapy improves survival and quality of life in advanced pancreatic and biliary cancer. *Ann Oncol* **7**: 593–600

Haie-Meder C, Pellae-Cosset B, Laplanche A, Lagrange JL, Tuchais C, Nogues C and Arriagada R (1993) Results of a randomized clinical trial comparing two radiation schedules in the palliative treatment of brain metastases. *Radiat Oncol* **26**: 111–116

Hopwood P, Stephens RJ and Machin D for the MRC Lung Cancer Working Party (1994) Approaches to the analysis of quality of life data: experiences gained from a Medical Research Council Lung Cancer Working Party palliative chemotherapy trial. *Quality Life Res* **3**: 339–352

James LE, Gower NH, Rudd RM, Spiro SG, Harper PG, Trask CW, Partridge M, Souhami RL and Ruiz de Elvira M-C (1996) A randomised trial of low-dose/high-frequency chemotherapy as palliative treatment of poor-prognosis small cell lung cancer: a Cancer Research Campaign trial. *Br J Cancer* **73**: 1563–1568

Kurtz JM, Gelber R, Brady LW, Carella RJ and Cooper JS (1981) The palliation of brain metastases in a favorable patient population: a randomized clinical trial by the Radiation Therapy Oncology Group. *Int J Radiat Oncol Biol Phys* **7**: 891–895

Leibel SA, Pajak TF, Massullo V, Order SE, Komaki RU, Chang CH, Wasserman TH, Phillips TL, Lipshutz J and Durbin LM (1987) A comparison of misonidazole sensitized radiation therapy to radiation therapy alone for the palliation of hepatic metastases: results of a Radiation Therapy Oncology Group randomized prospective trial. *Int J Radiat Oncol Biol Phys* **13**: 1057–1064

Lewington VJ, McEwan AJ, Ackery DM, Bayly RJ, Keeling DH, Macleod PM, Porter AT and Zivanovic MA (1991) A prospective randomised double-blind crossover study to examine the efficacy of strontium-89 in pain palliation in patients with advanced cancer metastatic to bone. *Eur J Cancer* **27**: 954–958

Maher EJ, Hopwood P, Girling DJ, Macbeth FR and Mansi JL (1994) Measurement of outcome in palliative oncology. *J Cancer Care* **3**: 94–102

Marty M, on behalf of the Granisetron Study Group (1992) A comparison of granisetron as a single agent with conventional combination antiemetic therapies in the treatment of cytostatic-induced emesis. *Eur J Cancer* **28A** (suppl. 1): S12–S16

Medical Research Council Lung Cancer Working Party (1989*a*) Survival, adverse reactions and quality of life during combination chemotherapy compared with selective palliative treatment for small cell lung cancer. *Respir Med* **83**: 51–58

Medical Research Council Lung Cancer Working Party (1989*b*) Controlled trial of twelve versus six courses of chemotherapy in the treatment of small cell lung cancer. *Br J Cancer* **59**: 584–590

Medical Research Council Lung Cancer Working Party (1991) Inoperable non-small cell lung cancer (NSCLC): a Medical Research Council randomised trial of palliative radiotherapy with two fractions or ten fractions. *Br J Cancer* **63**: 265–270

Medical Research Council Lung Cancer Working Party (1992) A Medical Research Council (MRC) randomised trial of palliative radiotherapy with two fractions or a single fraction in patients with inoperable non-small cell lung cancer (NSCLC) and poor performance status. *Br J Cancer* **65**: 934–941

Medical Research Council Lung Cancer Working Party (1993) A randomised trial of three or six courses of etoposide cyclophosphamide methotrexate and vincristine or six courses of etoposide and ifosfamide in small cell lung cancer (SCLC). II. Quality of life. *Br J Cancer* **68**: 1157–1166

Medical Research Council Lung Cancer Working Party (1996*a*) Randomized trial of palliative two-fraction versus more intensive 13-fraction radiotherapy for patients with inoperable non-small cell lung cancer and good performance status. *Clin Oncol* **8**: 167–175

Medical Research Council Lung Cancer Working Party (1996*b*) Randomized trial of four-drug versus less intensive two-drug chemotherapy in the palliative treatment of patients with small cell lung cancer (SCLC) and poor prognosis. *Br J Cancer* **73**: 406–413

Medical Research Council Lung Cancer Working Party (1996*c*) Comparison of oral etoposide and standard intravenous multidrug chemotherapy for small cell lung cancer: a stopped multicentre randomised trial. *Lancet* **348**: 563–566

Mohiuddin M, Chen E and Ahmad N (1996) Combined liver radiation and chemotherapy for palliation of hepatic metastases from colorectal cancer. *J Clin Oncol* **14**: 722–728

Muers MF and Round CE (1993) Palliation of symptoms in non-small cell lung cancer: a study by the Yorkshire Regional Cancer Organisation thoracic group. *Thorax* **48**: 339–343

Richards MA, Hopwood P, Ramirez AJ, Twelves CJ, Ferguson J, Gregory WM, Swindell R, Scrivener W, Miller J, Howell A and Rubens RD (1992) Doxorubicin and advanced breast cancer: influence of schedule on response, survival and quality of life. *Eur J Cancer* **28A**: 1023–1028

Stephens RJ, Girling DJ and Machin D (1994) Treatment-related deaths in small cell lung cancer: can patients at risk be identified? *Lung Cancer* **11**: 259–274

Sur RK, Kochhar R and Singh DP (1994) Oral sucralfate in acute radiation oesophagitis. *Acta Oncol* **33**: 61–63

Tannock IF, Osoba D, Stockler MR, Ernst DS, Neville AJ, Moore MJ, Armitage GR, Wilson JJ, Venner PM, Coppin CML and Murphy KC (1996) Chemotherapy with mitoxantrone plus prednisone or prednisone alone for symptomatic hormone-resistant prostate cancer: a Canadian randomised trial with palliative endpoints. *J Clin Oncol* **14**: 1756–1764

Tong D, Gillick L and Hendrickson FR (1982) The palliation of symptomatic osseous metastases. Final results of the study by the Radiation Therapy Oncology Group. *Cancer* **50**: 893–899

Twelves CJ, Dobbs NA, Lawrence MA, Ramirez AJ, Summerhayes M, Richards MA, Towlson KE and Rubens RD (1994) Iododoxorubicin in advanced breast cancer: a phase II evaluation of clinical activity, pharmacology and quality of life. *Br J Cancer* **69**: 726–731

Van Holten-Verzantvoort ATM, Kroon HM, Bijvoet OLM, Cleton FJ, Beex LVAM, Blijham G, Hermans J, Neijt JP, Papapoulos SE, Sleeboom HP, Vermey P and Zwinderman AH (1993) Palliative pamidronate treatment in patients with bone metastases from breast cancer. *J Clin Oncol* **11**: 491–498

World Health Organization (1979) *WHO Handbook for Reporting Results of Cancer Treatment*. WHO Offset publication no. 48. WHO: Geneva

*© Cancer Research Campaign 1999*
